# Impact of technological turbulence and competitive intensity on employee job performance: The role of workplace digitalization and job crafting

**DOI:** 10.1371/journal.pone.0330710

**Published:** 2025-08-18

**Authors:** Teng Liu, Cisheng Wu, Kexin Zhang, Manman Ge

**Affiliations:** School of Management, Hefei University of Technology, Hefei, Anhui Province, China; COMSATS University Islamabad - Wah Campus, PAKISTAN

## Abstract

Following the “environment-behavior-performance” paradigm, we aim to examine how the environment influences employee behavior and job performance. We subdivide the environment into the organizational external environment (dynamic environment, including technological turbulence and competitive intensity) and the specific internal work environment (workplace digitalization). Drawing on uncertainty reduction theory and conservation of resources theory, we validate the chain mediating mechanism through which technological turbulence and competitive intensity affect job performance via workplace digitalization and job crafting. The results indicate that technological turbulence has a significant direct positive effect on job performance (*b* = 0.233, *p* < .01). Additionally, it indirectly enhances job performance through a chain mediation pathway involving increased workplace digitalization and higher levels of avoidance crafting (*b* = 0.021, 95% CI [0.001, 0.056]). In contrast, the direct effect of competitive intensity on job performance is not statistically significant (*b* = 0.146, *p* = .126); however, it indirectly improves job performance through increased workplace digitalization and both approach crafting (**b* *= 0.121, 95% CI [0.064, 0.205]) and avoidance crafting (*b* = 0.051, 95% CI [0.022, 0.105]). Overall, this study applies a multi-level chain mediation model to provide empirical evidence that enriches and extends existing research on the effects of dynamic environments on employee job performance.

## Introduction

With the advancement of technology, companies are encountering growing environmental instability [1]. The environment serves as the objective condition for the survival of companies and maintains a close interactive relationship with organizations and individuals. The information conveyed by environmental changes is crucial for the development of both organizations and individuals [2,3], as all organizations and individuals operate within such a dynamic environment [4]. They would better pay close attention to the dynamic environment, which not only affects the strategic planning and management of the organization [5] but also has a great impact on the employees’ growth and development [2,3].

In response to digital transformation, companies are accelerating their digitalization efforts [6,7], which brings new challenges and opportunities for organizations and employees. Organizations and employees are expected to be responsive to digital transformation and actively use digital technologies [8] to better align themselves with the dynamic environment and ensure sustainable development. Workplace digitalization is the specific application of digital transformation that alters the way organizations operate and the design of employees’ work [2,9,10]. It has been established that employees in organizations with a high level of workplace digitalization can access resources more efficiently and maintain a high level of flexibility [8]. Accordingly, with the change of workplace digitalization, employees are best encouraged to craft their jobs to better adapt to the changes [11]. Employees may choose to actively interact with internal and external environments and adjust their behaviors to adapt to the surroundings [12]. For example, they may actively alter their tasks and roles and engage in bottom-up job design (i.e., job crafting) [13], thereby affecting job performance [14–16]. Job crafting of employees has proven to be closely linked to changes in internal and external environments [13]. For instance, the development of digital technology has made it possible for employees to be online anytime, anywhere, thanks to various mobile terminals. This means that employees need to embrace new ways of working and accept working in places other than the office [4]. These variations may significantly impact the employees’ work environment and add to uncertainty [4,17]. According to the uncertainty reduction theory and conservation of resources theory, when confronted with changes in external and internal organizational environments, employees are strongly motivated to upgrade their resources and naturally take proactive behaviors to mitigate the uncertainty they are exposed to [4], thereby maintaining high individual performance.

Several scholars have investigated the impact of organizational internal and external environments on employee behavior and outcomes. For instance, Song et al. [4] argued that dynamic environment significantly influences employees’ job crafting and performance. However, their research mainly focused on task crafting from the role-based perspective [18], without integrating the job demands-resources framework [13]. This limits our understanding of the multidimensional influence of dynamic environment on job crafting. Moreover, their study emphasized external environments while largely neglecting the role of specific internal organizational contexts. Additionally, Medici et al. [11] suggested that changes in external dynamic environments may prompt employees to engage in occupational crafting by actively or passively adopting various strategies to maintain career stability. In contrast, Slemp *et al.* [19] highlighted the critical role of internal organizational environments in shaping employee behavior and outcomes. Similarly, Hooi and Chan [7] found that workplace digitalization, an internal contextual factor, can effectively stimulate proactive employee behaviors. Nevertheless, limited research has examined how external dynamic environmental factors (e.g., technological turbulence and competitive intensity) and internal contextual factors (e.g., workplace digitalization) jointly influence employee job crafting and job performance. Against this background, we aim to address the following two research questions:

RQ1: How does the external dynamic environment (e.g., technological turbulence and competitive intensity) affect employee job performance through job crafting?

RQ2: What role does workplace digitalization play in the relationship between dynamic environment and job crafting? Does it, together with job crafting, form a chain mediation mechanism that affects employee job performance?

To address the above questions, and drawing on the “environment-behavior-performance” paradigm, we refine the environment by subdividing it into the external dynamic environment (technological turbulence and competitive intensity) and specific internal work context (workplace digitalization), we construct a chain mediation model (dynamic environment, workplace digitalization, job crafting, and job performance) to empirically reveal the internal transmission mechanism through which dynamic environment affect employee job performance. To the best of our knowledge, existing studies have not thoroughly examined the process by which technological turbulence and competitive intensity influence employee job performance via workplace digitalization and job crafting. Our study aims to fill this gap, providing novel theoretical insights and empirical evidence on how dynamic environment shapes employee behavior and performance.

## Literature review and hypotheses

### Dynamic environment and job performance

Dynamism is a critical characteristic of environments, which refers to a certain degree of rapid, unpredictable, and turbulent change [4,20]. As a shifting context, a dynamic environment is filled with both opportunities and challenges [21]. It compels employees to recognize environmental forces and reflect on the limitations of their own capabilities. In response, they conduct a comprehensive assessment and proactively adjust their behaviors to enhance adaptability [4]; e.g., proactively learning new knowledge and skills to maintain personal competitiveness and enhance job performance. Particularly in conditions of heightened technological turbulence and competitive intensity, the demands placed on employees increase significantly [4,22,23]. These include more frequent skill updates [7], greater demands for information processing and decision-making [10], and the need to adapt to evolving modes and intensity of social interaction [24]. These pressures lead employees to reassess how they engage with their work and environment, actively responding to and leveraging the opportunities and challenges presented by the dynamic environment.

We believe that technological turbulence and competitive intensity are two significant features of the dynamic environment [25]. Technological turbulence refers to the rate of technological updating and change [25,26]. With the advancement of digital technologies, such as ChatGPT, DeepSeek, and other OpenAI, the utility of the existing technology will decrease [4,27,28]. Therefore, the uncertainty of employees’ work environment is on the rise [1]. Based on the uncertainty reduction theory, employees tend to strengthen their capabilities [29] to reduce the anxiety caused by uncertainty [4]. The emergence of new technologies may impose higher demands on employees. In response, employees are strongly motivated to explore new technologies [30] and upgrade their resources [31] to maintain their core competencies, which ultimately helps improve their job performance. Therefore, we hypothesize:

H1. Technological turbulence positively affects employees’ job performance.

Competitive intensity refers to the degree of competitive pressure that a company faces in the market environment [26]. Research reveals that competition contributes to uncertainty in the industry, which not only affects access to resources at the company level [32] but also relates to the ease of employee access to resources [33]. When an employee realizes that resources are unable to meet demands, he/she may change his/her intentions and efforts [34] and improve the performance to match the new needs [35,36]. Especially when in an environment of high competitive intensity, employees are motivated to develop capabilities, explore new chances, and seek new resources [29]. Intensely competitive environments often require employees to possess more complex skills and to effectively acquire and apply new skills [37]. Doing so enables them to optimize work processes, reduce error rates, and enhance work efficiency [8,38,39], thereby improving their job performance [40]. Therefore, we hypothesize:

H2. Competitive intensity positively affects employees’ job performance.

### The mediating effect of workplace digitalization

The increasing prevalence of digital technologies provides new perspectives to organizations and management [36]. The application of digital technologies and tools is broadening the boundary of time and space in the workplace [4] to increase the adaptability of work. Workplace digitalization refers to organizational innovations where digital technologies and tools are introduced into the workplace [36] to change the mode of workplace operations and job design [2,10,41,42]. In a dynamic environment, employees are likely to maintain their competitiveness by intensifying the use of digital technologies and tools, which can help them attain a high degree of connectivity and flexibility. As a result, they can reduce the time and cost of obtaining information, increase productivity, and create more value [8].

The level of workplace digitalization is highly influenced by technological turbulence. The emergence of Artificial Intelligence, Blockchain, Cloud Computing, Big Data, and other “ABCD” technologies [43,44] has led to a constant updating of digital technologies [45], hence making them much more accessible and easier to use. In the workplace, employees can easily access and use the latest digital tools and digital channels, more simply and quickly free themselves from repetitive daily tasks [41], and have more time and energy for value-based creation. In addition, workplace digitalization blurs the boundaries of space and time among employees [10,46] (e.g., the normalization of telecommuting and flexible working, a new office style that frees employees from the constraints of the traditional office and working time [4], and allows them to deal with their work anytime and anywhere), increasing work convenience and flexibility [36], and improving work efficiency and job performance while reducing work costs [47]. Therefore, we hypothesize:

H3. Workplace digitalization mediates the relationship between technological turbulence and job performance.

Companies are facing a complex and changing competitive environment. Competitive stresses are motivating companies to engage in digital transformation [7,48]. Because digital transformation is one of the main trends and challenges in the development of current companies [49], it can help companies maintain their competitiveness and improve their adaptability to the environment. In detail, the employment of digital technologies and tools contributes to the digital capabilities of companies and reduces competitive stress. And, the enhancement of digital capabilities helps companies build a platform for information interaction, which accelerates the flow of Information, reduces the probability of employee errors and mistakes, and ultimately increases employee efficiency and corporate competitiveness [6,38,50].

The impact of workplace digitalization varies within different industries [51]. As digital technologies and organizational infrastructures are renewed, workplace digitalization has a greater effect on employees with high-skilled job requirements, which may lead to a higher intensity of competition [52]. Consequently, to remain competitive, these employees need to continually learn new digital technologies and utilize new digital tools to meet job requirements, complete work tasks quickly, and improve work efficiency [53]. Therefore, we hypothesize:

H4. Workplace digitalization mediates the relationship between competitive intensity and job performance.

### The mediating effect of job crafting

Job crafting is defined as proactive change by employees to optimize their demands and resources to better fit their jobs to improve their well-being and performance [54]. Zhang and Parker [13], based on the three-level hierarchical structure of orientation-form-content, define two forms of job crafting: approach crafting (i.e., effortful and directed actions to seek positive aspects of work) and avoidance crafting (effortful and directed actions to avoid, or escape from, negative aspects of work). Building on Zhang and Parker [13], Tims et al. [54], and Fong et al. [55]. We apply the categorization of approach and avoidance crafting in the following ways: approach crafting represents increasing structural job resources, increasing social job resources, and increasing challenging job demands, while decreasing hindering job demands is a proxy for avoidance crafting [55–57].

With the rapid advancement of digital technologies, employees are increasingly finding their existing knowledge and resources insufficient to cope with emerging task requirements due to intensified technological turbulence [45]. Drawing upon conservation of resources theory, employees are motivated to enhance their resource reservoirs to preserve self-worth and social relationships [31], thereby exhibiting a greater propensity to engage in job crafting behaviors to adapt to environmental changes [30]. Specifically, employees may adopt approach crafting to proactively develop new technologies, optimize work methods, and acquire additional resources [30,38,58], consequently enhancing work engagement and job performance. Especially when employees perceive increasing technological pressure and heightened uncertainty [7,10,46], approach crafting can help enhance their positive experiences and capability development [2,13]. Conversely, avoidance crafting embodies employees’ motivation to regulate stress and fatigue by intentionally reducing task demands and emotional strain [55,59]. This strategy enhances perceived work manageability and sustainability, which may, in turn, indirectly facilitate job performance [14]. Although approach and avoidance crafting are driven by different motivational forces, both represent adaptive responses to technological change [16,57]. Prior research has shown that job crafting can boost employees’ ability to acquire resources and achieve task-person fit [60], thereby playing a crucial mediating role between technological turbulence and job performance. Accordingly, whether aimed at personal growth or stress alleviation, both forms of job crafting serve as important mechanisms through which employees navigate technological turbulence and achieve sustainable job performance. Therefore, we hypothesize:

H5. Job crafting mediates the relationship between technological turbulence and job performance, including approach crafting and avoidance crafting.

The development and application of digital technologies have intensified the competition faced by companies [61,62]. On the one hand, to meet organizational expectations and maintain individual competitiveness, employees often engage in approach crafting by proactively adjusting task content, acquiring new skills, and adopting new tools, thereby enhancing the challenge and creativity of their work [45] and ultimately improving adaptability and job performance [16]. On the other hand, competitive pressure may trigger employees’ anxiety about meeting high performance standards amid limited resources [63], heightening their perception of uncertainty [7,64]. In this context, some employees tend to engage in avoidance crafting by reducing high-demand tasks or maintaining existing work routines to alleviate psychological strain [4,65]. Notably, while pursuing high performance, organizations may simultaneously provide employees with additional support resources and development opportunities to guide employees in reallocating resources and reframing tasks [66], thereby mitigating the sense of maladaptation induced by changes in competitive intensity. Collectively, job crafting functions as a proactive adaptation strategy through which employees respond to competitive environments, serving as a key mediating mechanism between competitive intensity and job performance. Specifically, approach crafting helps stimulate employee potential and enhance job performance, while avoidance crafting assists in stress regulation and may indirectly contribute to job performance. Therefore, we hypothesize:

H6. Job crafting mediates the relationship between competitive intensity and job performance, including approach crafting and avoidance crafting.

### The chain mediating effect of workplace digitalization and job crafting

In recent studies, it has been revealed that digital change in the workplace affects employee behavior [2,8–10,67]. The application of new technologies and tools makes work unpredictable and unstable [8]. The awareness and behavioral adjustments made by employees, who are the main executors of work, in the face of digital change are crucial to the survival and development of individuals and organizations [68]. When employees adopt approach crafting behaviors in response to digital change, they are more likely to proactively acquire digital knowledge, apply digital tools, and enhance their digital literacy to increase their personal resource levels. Conversely, workplace digitalization may also trigger fear and anxiety among employees [10,69], who are concerned about being replaced by advanced technology [40] and therefore adopt avoidance crafting behaviors toward digital change. They may prefer to reduce work complexity or workload intensity [55] and make conscious adjustments to their demands and resources. As a result, workplace digitalization is highly effective in triggering job crafting for employees.

Based on the “environment-behavior-performance” paradigm [4,70], when in an environment of high technological turbulence, digital tools and channels in the workplace are more easily accessible, and the use of digital technologies is more prevalent. For employees, this means simpler access to resources and fulfillment of demands, which is conducive to improving productivity and efficiency [40]. When in a highly competitive intensity environment, companies are more oriented towards digital change for sustainable growth, and thus, the adoption of digital technologies in the workplace will be higher. This will have an impact on the digital skills of employees [71] because, to enhance their value and remain competitive in employment, employees will constantly adjust their working ways in practice to satisfy the demands of digital transformation. Improvements in digital skills mean that employees can complete tasks faster, communicate and collaborate more easily, record and manage information more clearly, and so on. Therefore, we hypothesize:

H7a. Workplace digitalization and approach crafting play a chain mediating effect between technological turbulence and job performance.

H7b. Workplace digitalization and avoidance crafting play a chain mediating effect between technological turbulence and job performance.

H8a. Workplace digitalization and approach crafting play a chain mediating effect between competitive intensity and work performance.

H8b. Workplace digitalization and avoidance crafting play a chain mediating effect between competitive intensity and work performance.

**Fig 1:**
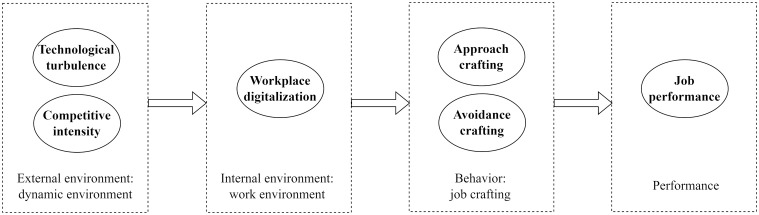
Theoretical model.

According to the literature review and hypothesis development, we construct a conceptual model as presented in Fig 1.

## Methods

### Participants

Data for this study were collected from June to October 2023 using a combination of convenience sampling and purposive sampling. Part of the sample consisted of working MBA students, while the other part was obtained through field surveys conducted in selected companies across industries such as pharmaceuticals, technology, and automotive manufacturing. All participants were full-time employees distributed across multiple cities in Anhui Province, China. Although paper-based questionnaires have certain limitations in terms of convenience, prior research has suggested that they tend to yield slightly higher response rates than web-based surveys [72,73]. Based on this, this study employed paper questionnaires for data collection. A total of 740 questionnaires were distributed through offline channels, and 569 were returned, resulting in a response rate of 76.89%. After excluding 123 invalid responses—due to missing data, straight-lining, or incorrect answers to attention-check items (e.g., “To confirm you are answering this questionnaire seriously, please select option 3”)—a total of 446 valid questionnaires were retained, yielding a final validity rate of 78.38%.

Our participants (51% male, $M_{age}$ = 32.35, ${SD}_{age}$ = 6.81) work an average of 46.89 hours per week (SD = 20.98) and have been working for their current company for 5.82 years (SD = 6.38). They are also generally well educated, with 91.26% having a bachelor’s degree or higher. Participants are employed in various industries, such as construction (31%), real estate (18%), manufacturing (12%), agriculture, forestry, and fisheries (4%), finance and insurance (4%), social services (3%), and information technology (2%).

### Ethics statement

This study was approved for exemption from ethical review by the institutional ethics committee. Participation was entirely voluntary, and only individuals who gave explicit verbal consent were invited to take part in the study. Prior to participation, we clearly informed all potential respondents that the questionnaires were solely for academic research purposes and would be conducted anonymously, with strict confidentiality maintained for all information provided.

### Measurement instruments

All scales are translated from English to Chinese using the translation-back-translation procedure proposed by Brislin [74]. A five-point Likert scale is adopted for all, ranging from 1 for strongly disagree to 5 for strongly agree.

### Dynamic environment

We measure technological turbulence mainly based on Jaworski and Kohli’s [25] scale. Technological turbulence is measured with four items (e.g., “the technology in our industry is changing rapidly, and the continuous emergence and application of new digital technology accelerate the speed of change.”). Cronbach’s α is 0.88. Competitive intensity is measured with four items (e.g., competition in our industry is cutthroat, and the development and application of digital technology has intensified competition). To better align with the digital context, we add a digital technology background to each item (e.g., we change “Technological changes provide big opportunities in our industry” to “Technological changes provide big opportunities in our industry, and the application of digital technology enhances the competitiveness of products and services.”). Cronbach’s α is 0.76.

### Workplace digitalization

Workplace digitalization is measured with fourteen items of the scale provided by Ouyang et al. [8]. The scale is divided into four subscales, including performance expectancy (e.g., using the digital technology enables me to accomplish tasks more quickly; three items; Cronbach’s α is 0.90), effort expectancy (e.g., learning to use the digital technology would be easy for me; four items; Cronbach’s α is 0.87), and social influence (e.g., I use the digital technology because of the proportion of coworkers who use the digital technology; four items; Cronbach’s α is 0.80), and facilitating conditions (e.g., a specific person (or group) is available for assistance with difficulties in using digital technology; three items; Cronbach’s α is 0.70).

### Job crafting

Approach and avoidance crafting is measured using the Job Crafting Scale developed by Tims et al. [54]. Approach crafting consists of fifteen items divided into 3 subscales, including increasing structural job resources (e.g., I try to learn new things at work, especially about digital technology; five items; Cronbach’s α is 0.89), increasing social job resources (e.g., I ask others for feedback on my job performance; five items; Cronbach’s α is 0.84), and increasing challenging job demands (e.g., I regularly take on extra tasks even though I do not receive extra salary for them; five items; Cronbach’s α is 0.83). Avoidance crafting consists of five items and is measured using the subscale (decreasing hindering job demands) (e.g., I try to ensure that my work is emotionally less intense; five items; Cronbach’s α is 0.85). We removed one item (i.e., I organize my work in such a way to make sure that I do not have to concentrate for too long a period at once) because employees in the field study reported difficulty answering this question.

### Job performance

Job performance is measured using a five-item scale developed by Williams and Anderson [75–77]. For example, I can meet formal performance requirements of the job. Cronbach’s α is 0.90.

### Control variables

Based on existing studies, gender [78] and age [79] can affect employees’ perceptions and reactions to the workplace. Also, employees with longer tenure and higher levels of education are believed to have accumulated more work and general knowledge, making them potentially better able to engage in job crafting in the face of environmental changes [80]. And, employees who have longer working hours typically have more opportunities to engage in job crafting [81]. Thus, we select the five demographic and job-related variables, namely gender, age, tenure, weekly working hours, and education, as control variables [57,82]. The results indicate that employees’ tenure and age are significantly correlated with certain variables. However, including these factors in the structural equation model does not alter the main findings. Therefore, we retain the simpler model.

### Common method bias test

Since the self-assessment method may lead to the issue of common method bias (CMB) among variables, we first do Harman’s single-factor test, which is one of the simplest and most effective methods for CMB issues [83]. Conducting a principal component analysis on all items, we obtain 10 factors with eigenvalues greater than 1 without specifying the number of factors to extract. Among them, the factor with the largest eigenvalue, when not rotated, explains a variance of 31.86%, which represents that it is within the reasonable allowable range and can be used for subsequent data analysis. To further assess CMB, a control variable with low correlation to the main constructs serves as a proxy variable and is controlled for all latent variables. All regression paths are non-significant, indicating that CMB is unlikely to be a serious concern in this study.

## Results

### Confirmatory factor analyses

We employ Confirmatory Factor Analysis (CFA) in Mplus to test the construct validity. The results are summarized in Table 1, which proposes that the six-factor model (technological turbulence, competitive intensity, workplace digitalization, approach crafting, avoidance crafting, and job performance) is optimal among all the alternative models ($\chi^{2}/df$ = 2.083, **p* *< .01; RMSEA = 0.049, CFI = 0.910, TLI = 0.904, SRMR = 0.057). This suggests that the discriminant validity of the six-factor model is more satisfactory. Also, we test measurement invariance across gender, and the model shows good fit and cross-gender consistency.

**Table 1:** CFA results.

Model	Factor	$\chi^{2}/df$	RMSEA	CFI	TLI	SRMR
6-factor^a^	TT;CI;WD;APJC;AVJC;JP	2.083	0.049	0.910	0.904	0.057
5-factor	TT + CI;WD;APJC;AVJC;JP	2.265	0.053	0.894	0.887	0.061
4-factor	TT + CI;WD;APJC+AVJC;JP	3.069	0.068	0.825	0.816	0.066
3-factor	TT + CI + WD;APJC+AVJC;JP	4.712	0.091	0.685	0.669	0.079
2-factor	TT + CI + WD+APJC+AVJC;JP	5.625	0.102	0.606	0.588	0.093
1-factor	TT + CI + WD+APJC+AVJC+JP	6.628	0.112	0.521	0.499	0.099

Notes: TT is technological turbulence; CI is competitive intensity; WD is workplace digitalization; APJC is approach crafting; AVJC is avoidance crafting; and JP is job performance. “+” is factor merging.

The hypothesized model of 6-factor does not reach acceptable fit ($\chi^{2}/df$ = 2.135, *p* < .01, RMSEA = 0.050, CFI = 0.905, TLI = 0.899, SRMR = 0.058).

^a^Based on the suggestions of modification indices, two items of AVJC conditions are indicated as correlated with each other. We then report this final result in the table.

### Descriptive statistics and correlations

Table 2 reports the descriptive statistics of and correlations among the variables. Consistent with our expectations, technological turbulence is significantly positively correlated with job performance (**r* *= 0.359, **p* *< .01), and competitive intensity is significantly positively correlated with job performance (**r* *= 0.315, **p* *< .01), which provides preliminary support for H1 and H2.

**Table 2:** Correlations, means, and standard deviations of study variables.

	M	SD	1	2	3	4	5	6	7	8	9	10	11
1. TT	3.950	0.777	–										
2. CI	3.730	0.741	0.631^**^	–									
3. WD	3.593	0.610	0.429^**^	0.460^**^	–								
4. APJC	3.697	0.627	0.410^**^	0.428^**^	0.603^**^	–							
5. AVJC	3.815	0.679	0.309^**^	0.321^**^	0.375^**^	0.622^**^	–						
6. JP	4.042	0.652	0.359^**^	0.315^**^	0.375^**^	0.546^**^	0.484^**^	–					
7. Age	32.350	6.809	0.004	0.063	0.116^*^	0.053	0.024	0.005	–				
8. Gender	1.490	0.500	−0.067	0.022	−0.051	−0.065	0.010	−0.037	−0.151^**^	–			
9. Education	3.240	0.619	0.012	0.026	−0.064	0.007	−0.014	0.046	−0.079	−0.111^*^	–		
10. Tenure	5.820	6.378	0.039	0.097^*^	0.178^**^	0.089	0.046	0.085	0.758^**^	−0.036	−0.095^*^	–	
11. Hour	46.890	20.984	−0.022	0.001	0.000	−0.003	−0.012	−0.009	−0.007	−0.119^*^	0.188^**^	0.013	–

Notes: TT is technological turbulence; CI is competitive intensity; WD is workplace digitalization; APJC is approach crafting; AVJC is avoidance crafting; and JP is job performance. * **p* *< .05; ** **p* *< .01. Two-tailed.

### Hypotheses testing

#### Main effect.

We construct SEM 1 to test the effect of technological turbulence and competitive intensity on job performance in AMOS. The proposed model 1 presents an acceptable fit to the data ($\chi^{2}/df$ = 2.505, **p* *< .01; RMSEA = 0.058, CFI = 0.972, TLI = 0.96, SRMR = 0.031), verifying the rationality of the model.

In SEM 1, there is a significantly positive association between technological turbulence and job performance (**b* *= 0.233, **p* *< .01), indicating that technological turbulence can improve employees’ job performance. Thus, H1 is supported. Competitive intensity has a positive effect on job performance (**b* *= 0.146, **p* *= .126), while the effect is not statistically significant. Thus, H2 does not receive empirical support.

#### Mediating effect.

Because the specific indirect effects cannot be tested in SEM, we used phantom models to test our mediation hypotheses [84]. Outside of the main SEM, the extra latent variable is placed with equal variances to the corresponding variables, and equality constraints are set (the path coefficients between phantom variables are equal to the corresponding path coefficients in the main SEM). This step allows us to test for specific mediating effects by generating 95% bootstrapped confidence intervals for each indirect effect [84].

Based on the above analysis, the first procedure is to use workplace digitalization and job crafting as independent mediating variables to construct SEM 2 (technological turbulence, competitive intensity, workplace digitalization, and job performance) ($\chi^{2}/df\;$= 2.623, **p* *< .01; RMSEA = 0.060, CFI = 0.927, TLI = 0.918, SRMR = 0.052) and SEM 3 (technological turbulence, competitive intensity, approach crafting, avoidance crafting, and job performance) ($\chi^{2}/df$ = 2.423, **p* *< .01; RMSEA = 0.057, CFI = 0.916, TLI = 0.909, SRMR = 0.045). The models show acceptable data fit, validating the models.

In SEM 2, the indirect effect between technological turbulence and job performance is not significant (**b* *= 0.034, 95%CI [−0.005, 0.092]), indicating that workplace digitalization does not play a significant mediating role in the relationship between technological turbulence and job performance. Thus, H3 is rejected. The indirect effect between competitive intensity and job performance is significant (**b* *= 0.120, 95%CI [0.054, 0.232]), meaning that workplace digitalization significantly mediates the relationship between competitive intensity and job performance. Thus, H4 is supported.

In SEM 3, the indirect effect mediated by approach crafting (**b* *= −2.925, 95%CI [−23.516, −0.509]) and avoidance crafting (**b* *= −1.009, 95%CI [−13.508, −0.076]) is significant between technological turbulence and job performance, suggesting that job crafting plays a significant mediating role in the relationship between technological turbulence and job performance. Thus, H5 is supported. Namely, job crafting produces a negative effect on the relationship between technological turbulence and job performance. The mediating indirect effects of approach crafting (**b* *= 4.965, 95%CI [1.049, 34.991]) and avoidance crafting (**b* *= 1.646, 95%CI [0.177, 17.947]) are significant between competitive intensity and job performance, indicating that job crafting significantly mediates the relationship between competitive intensity and job performance. Thus, H6 is supported. The summary of SEM 2 and SEM 3 results is presented in Table 3.

**Table 3:** The summary of the results for SEM 2 and SEM 3.

	Structural Equation Paths (Indirect Effect)	Effect	SE	95%CI Lower	95%CI Upper	P
SEM 2	TT → WD → JP	0.034	0.024	−0.005	0.092	0.081
	CI → WD → JP	**0.120**	0.043	0.054	0.232	0.000
SEM 3	TT→APJC→JP	**−2.925**	3.844	−23.516	−0.509	0.017
	TT→AVJC→JP	**−1.009**	2.054	−13.508	−0.076	0.027
	CI→APJC→JP	**4.965**	5.890	1.049	34.991	0.002
	CI→AVJC→JP	**1.646**	2.933	0.177	17.947	0.010

Note: TT is technological turbulence; CI is competitive intensity; WD is workplace digitalization; APJC is approach crafting; AVJC is avoidance crafting; and JP is job performance; “→” is path direction. Bold values indicate significant results.

**Fig 2:**
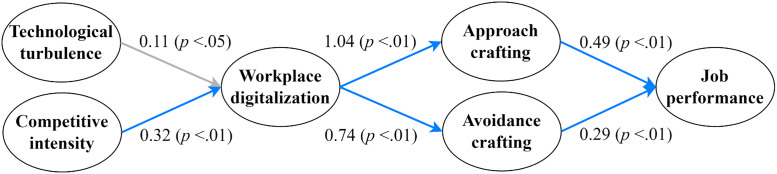
Estimated path coefficients among key variables. Notes: Unstandardized path coefficients derived from structural equation modeling. Arrows in blue indicate significant positive paths (*p* < .01); arrows in grey indicate significant positive paths (*p* < .05).

Furthermore, the next procedure is to test the chain mediating effects of workplace digitalization and job crafting. Fig 2 presents the path model of the effects of technological turbulence and competitive intensity on job performance via workplace digitalization and job crafting, along with a summary of the results.

The results demonstrate that technological turbulence (**b* *= 0.11, **p* *< .05) and competitive intensity (**b* *= 0.32, **p* *< .01) are positively associated with workplace digitalization, workplace digitalization is positively associated with approach crafting (**b* *= 1.04, **p* *< .01) and avoidance crafting (**b* *= 0.74, **p* *< .01), and approach crafting (**b* *= 0.49, **p* *< .01) and avoidance crafting (**b* *= 0.29, **p* *< .01) are positively associated job performance, which provide preliminary support for H7a, H7b, H8a and H8b.

To examine the chain mediating role of workplace digitalization and job crafting in the relationship between the two types of dynamic environment and job performance, we investigate the phantom models. The results are shown in Table 4, which indicates that the indirect effect of workplace digitalization and approach crafting between technological turbulence and job performance is not significant (**b* *= 0.050, 95%CI [−0.002, 0.113]); in other words, the chain mediating effect is not valid. Thus, H7a is rejected. The indirect effect of workplace digitalization and avoidance crafting between technological turbulence and job performance is significant (**b* *= 0.021, 95%CI [0.001, 0.056]), meaning that the chain mediating effect is valid. Thus, H7b is supported.

**Table 4:** The summary of indirect effect results in phantom models.

Structural Equation Paths (Indirect Effect)	Effect	SE	95%CI Lower	95%CI Upper	P
TT → WD→APJC→JP	0.050	0.029	−0.002	0.113	0.054
TT → WD→AVJC→JP	**0.021**	0.013	0.001	0.056	0.035
CI → WD→APJC→JP	**0.121**	0.035	0.064	0.205	0.000
CI → WD→AVJC→JP	**0.051**	0.020	0.022	0.105	0.000

Note: TT is technological turbulence; CI is competitive intensity; WD is workplace digitalization; APJC is approach crafting; AVJC is avoidance crafting; and JP is job performance; “→” is path direction. Bold values indicate significant results.

The indirect effect is significant in the relationship among competitive intensity, workplace digitalization, approach crafting (**b* *= 0.121, 95%CI [0.064, 0.205])/ avoidance crafting (**b* *= 0.051, 95%CI [0.022, 0.105]), and job performance, that is, the chain mediating effects are valid. Thus, H8a and H8b are supported.

## Conclusion

### Research conclusion

We found that dynamic environment affects employee job performance. Among them, a) technological turbulence has a significant positive effect on employee job performance. This finding indicates that the development of digital technology may have optimized workflow and made information acquisition and processing more convenient [8], thereby reducing employees’ work burden and improving work efficiency. The direct effect of competitive intensity on employee job performance is not significant, which may be due to offsetting positive and negative influences. On the one hand, a competitive environment may enhance organizational goal orientation and a sense of urgency; on the other hand, it can increase employee stress and deplete resources. Additionally, competitive intensity may primarily affect job performance through indirect pathways, thereby diminishing its direct effect; b) in the indirect relationship between technological turbulence and job performance, workplace digitalization does not play a separate mediating role. The probable reason is that workplace digitalization fails to fully capture the mediating effect between technological turbulence and job performance. Independent job crafting (i.e., approach crafting and avoidance crafting) negatively mediates the positive relationship between technological turbulence and job performance. Technological turbulence may exacerbate employees’ uncertainty and anxiety regarding future tasks [7], prompting reactive, coping-oriented job crafting [10]. However, such job crafting is often a passive response rather than a proactive effort aimed at personal growth, which can lead to resource depletion and increased psychological burden, thereby impairing job performance; c) as expected, we empirically find that workplace digitalization can act as a chain mediator via the path of avoidance crafting. A possible reason is that technological advancements accelerate the digitalization process of companies and improve the level of workplace digitalization, which in turn may affect the way employees perform. In this context, employees may avoid or reduce negative work tasks and interpersonal relationships [55] to maintain a positive mindset and good working conditions [16]. Moreover, considering the analysis above, there may be an offsetting effect of workplace digitalization and approach crafting, thus the chain mediating effect via the path of approach crafting is not significant; d) in the indirect relationship between competitive intensity and job performance, both workplace digitalization and job crafting (i.e., approach crafting and avoidance crafting) act as separate positive mediators, and workplace digitalization exerts a chain mediating effect through job crafting. This may be mainly because, in the context of digitalization, the increasing intensity of competition in the market will prompt companies to accelerate the process of digital transformation [48], resulting in the enhancement of their digital capabilities. As a natural consequence, the enhancement of digital capabilities makes it easier to provide employees with more resources and development opportunities [46], such as the flexibility to deliver more online training regardless of time and location. Based on this, employees are more likely to take proactive measures to increase challenging work tasks and avoid or reduce negative work tasks, thus boosting their well-being and performance at work.

### General discussion

Studies related to predictors of job crafting have mainly been carried out from the perspectives of the crafter’s characteristics [85–87], social relationships [55,76,88], and organizational culture [89–91], ignoring the effect of dynamic environments external to the organization (e.g., digital change motivates employees to improve their digital literacy) on the specific work environment as well as employees‘ behaviors within the organization. Unlike studies confined to organizational-level environments, our study introduces both the external digital environment and specific internal work environment into the model to examine the impact of the dynamic environment on workplace digitalization and employee responses from the perspectives of technological turbulence and competitive intensity. Compared with prior research, existing studies based on data from countries such as Switzerland, Australia, and the Netherlands have primarily focused on how internal organizational factors affect job crafting and employee job performance, with relatively limited attention to the mechanisms through which external macro-environmental factors exert influence [11,19,91]. Our findings suggest that intensified technological change and competitive intensity drive organizations to pursue digital transformation, which in turn stimulates employees to engage in job crafting as a means of adapting to new demands and enhancing their performance. Drawing on a field study, this study further reveals how external and internal environmental factors interact to shape employee behavior and outcomes.

### Theoretical implications

Based on the “environment-behavior-performance” paradigm [4,70], we rethink the relationship between the dynamic environment and employees’ job crafting behaviors and performance. Moreover, we expand the existing perspective that “a single environmental context influences employee job crafting and job performance” [4]. Most of the current studies consider job crafting as a “contextualized” activity that is influenced by the characteristics of the environment [13]. Therefore, the dynamic environment does have an impact on employees’ job crafting and performance. However, it overlooks the specific causes of employee behavioral changes in the environment. This study takes the context of digital technology and economic development into account, subdividing the dynamic environment into two scenarios: technological turbulence and competitive intensity, and investigating their effects on job crafting and performance, respectively. The results demonstrate that both technological turbulence and competitive intensity have an impact on employees’ job crafting, but the effects are not identical. This is consistent with the view that different contexts can affect job crafting to various extents [18].

Moreover, prior research suggests that external environments profoundly shape the work environment faced by employees [8,17] and that employees are capable of perceiving and responding to such environmental changes [92]. Given that work environment influences employees’ behavior [8], we introduce a critical internal organizational context, workplace digitalization, as a mediating variable and construct a chain mediation model (i.e., dynamic environment – workplace digitalization – job crafting – job performance). This model reveals how the external environment influences employee job performance via internal organizational context and individual adaptive behaviors, thereby offering a clearer view into the mechanisms linking environment and employee outcomes. In particular, it sheds light on how digitalized work contexts shape employees’ adaptive responses, helping to unpack the “black box” between dynamic environment and employee job performance.

Overall, by identifying both external and internal environmental antecedents of job crafting, along with its outcome variables, this study extends the theoretical boundaries of job crafting.

### Limitations and future research

Several limitations of this study ought to be discussed. Several limitations of this study warrant discussion. First, we collect data through self-report measures to more accurately capture employees’ responses to environmental changes, which helps address the covert nature of job crafting. As a form of behavioral adjustment involving autonomous job redesign, job crafting reflects subjective experiences and internal motivations that are not easily observed or recorded by others. Self-assessments allow employees to directly report their behavioral changes, thereby providing detailed first-hand information that enhances data accuracy. Harman’s single-factor test shows that CMB remains within acceptable limits. Nevertheless, future research may adopt multi-source data collection approaches, such as peer-reported job crafting, as coworkers can often observe certain aspects of a colleague’s behavioral changes [55,57]. Furthermore, we use cross-sectional data to examine the impact of dynamic environments on job performance via workplace digitalization and job crafting. Although each participant is given at least two weeks to complete the questionnaire, this timeframe may still be insufficient to capture potential longitudinal effects. Longitudinal and experimental approaches have been suggested to address such limitations and enhance the robustness of findings. Therefore, future research could consider adopting experimental or longitudinal designs to better understand the causal mechanisms involved. Moreover, as the study is conducted within a Chinese context, the generalizability of the findings may be limited. Given the cultural differences in values and organizational behaviors, such as the emphasis on collectivism in China versus individualism in Western societies, future research could employ cross-cultural comparative studies to enhance external validity and broaden the applicability of the results.

Also, this study focuses on individual behavioral responses to internal and external environments, without examining the interpersonal dynamics of job crafting within teams. Future research could explore how employees’ approach and avoidance crafting influence coworkers’ perceptions and behaviors, and how such interactions shape individual and team performance at the collective level. Another limitation of this paper is that it does not examine boundary conditions. The pathways of the dynamic environment on the behavior and outcomes of job crafting may be influenced by situational factors and individual differences [4,56]. Future research could consider the impact of organizational climate, regulatory focus, crafter characteristics, and so on. For instance, when facing environmental changes, employees may proactively alter themselves and engage in continuous self-regulation to achieve a better person-job fit. Hence, regulatory focus may affect employees’ perceptions of and responses to dynamic environments [4]. Future research can delve deeper into the behavioral choices and outcomes of crafters under the effect of different types of regulatory focus.

### Practical implications

First, our findings suggest that dynamic environment, characterized by technological turbulence and competitive intensity, not only affects the pace of organizational digital transformation but also significantly influences employees’ job crafting behaviors and job performance. Organizations are expected not to regard such environments merely as sources of pressure. Instead, they should recognize their potential to stimulate proactive adaptation among employees and teams.

Second, when faced with a dynamic environment, organizations need to seize opportunities to embrace digital transformation in line with current trends. Workplace digitalization can enhance work efficiency, flexibility, and the ease of resource acquisition [36,41]. Managers are encouraged to promote digitalization by providing adequate digital infrastructure and necessary support, enabling employees to integrate digital tools effectively into their daily work.

Third, job crafting serves as an effective strategy for employees to cope with change. Organizations should offer a work environment that fosters both skill development and employee autonomy. Specifically, granting employees an appropriate level of independence can encourage approach crafting behaviors, such as taking initiative or seeking feedback. When avoidance crafting behaviors emerge, such as efforts to reduce task complexity, organizations should respond promptly with interventions, including positive feedback and incentive mechanisms, to guide employees toward focusing on their strengths and long-term development goals.

## Supporting information

S1 Data (research data). (XLSX)
